# Comparative Analysis of the Efficacy of 1% Tropicamide and 1% Cyclopentolate Eye Drops for Cycloplegic Refraction in Indonesian Paediatric Population

**DOI:** 10.22599/bioj.491

**Published:** 2026-02-11

**Authors:** Randy Sarayar, Dian Estu Yulia, Tri Rahayu, Aria Kekalih

**Affiliations:** 1 Universitas Indonesia, Indonesia; 2 Department of Ophthalmology, Faculty of Medicine, Universitas Indonesia, Dr. Cipto Mangunkusumo National General Hospital, Jakarta, Indonesia; 3 Department of Community Medicine, Faculty of Medicine, Universitas Indonesia, Dr. Cipto Mangunkusumo National General Hospital, Jakarta, Indonesia

**Keywords:** cycloplegia, accommodation amplitude, refraction examination, tropicamide 1%, cyclopentolate 1%

## Abstract

**Background:**

Cycloplegic agents are essential for paediatric refraction examinations due to strong accommodative power in children. Accurate cycloplegia is crucial to avoid misdiagnosis of refractive errors such as latent hyperopia or pseudomyopia. Prior studies indicate that dark irises may require higher doses and exhibit a prolonged onset compared to lighter irises. Given that most Indonesians have brown irises, a comparative study of cycloplegic agents is necessary to optimise clinical application.

**Methods:**

This randomised, double-blind, controlled non-inferiority trial compared the residual accommodation amplitude (AA) following two drops of 1% Tropicamide (TRP1%) or 1% Cyclopentolate (CYC1%), administered 5 min apart. Each participant underwent both regimens in separate sessions one week apart. AA was measured using the NIDEK ARK-1s autorefractor, and side effects were assessed through interviews.

**Results:**

Seventy children (median age: 11 [9–12] years) participated. Both eyes were analysed separately. The eyes analysed were mostly myopic (70%) and emmetropic (26.43%). AA analysis showed no significant differences between TRP1% and CYC1% at 20, 30, and 40 min post-administration (*p* > 0.05), with maximum cycloplegic effect achieved before 20 min. Side effects were significantly higher with CYC1% than TRP1% (53.33% vs. 23.33%, *p* < 0.001).

**Conclusion:**

Tropicamide 1% is non-inferior to cyclopentolate 1% for cycloplegia in Indonesian children with brown irises. Given its comparable efficacy and lower incidence of side effects, Tropicamide 1% may serve as a safer alternative for paediatric cycloplegic refraction. Further studies with larger samples are warranted to refine dosing strategies and enhance clinical outcomes.

## Introduction

Cycloplegic refraction is a critical component of paediatric ophthalmic examinations, as it neutralises the strong accommodative response in young children, allowing for more accurate assessment of refractive errors. Evidence suggests that non-cycloplegic refraction tends to overestimate myopia and underestimate hyperopia, leading to inappropriate prescriptions that may contribute to the progression of myopia, amblyopia, or strabismus (Wilson *et al*., 2022). Twelker *et al*. reported that cycloplegic refraction using cyclopentolate results in an average increase of 0.50 to 0.75 dioptres in hyperopic measurements compared to non-cycloplegic retinoscopy, underscoring the necessity of cycloplegia in refractive evaluations (Twelker and Mutti, 2001). Given that myopia progresses most rapidly between the ages of 7 and 10, precise refraction during this critical period is essential to mitigating the increasing burden of myopia in Indonesia.

Cyclopentolate and tropicamide are the two primary cycloplegic agents used in clinical practice, with cyclopentolate providing a more potent effect but also associated with prolonged visual blur and discomfort that may be attributed to its longer duration on accommodation paralysis (24–48 h cyclopentolate vs. 6–7 h tropicamide) (Chmielarz-Czarnocińska and Gotz-Więckowska, 2022; Pei *et al*., 2021). While studies on predominantly Caucasian populations suggest that both agents are similarly effective (Al-Thawabieh *et al*., 2024; Doherty *et al*., 2019; Doyle, O’Dwyer and Harrington, 2023; Kyei *et al*., 2017; Lovasik, 1986; Siu *et al*., 1999), their efficacy in populations with darker irises, such as the Indonesian paediatric population, remains insufficiently studied. The American Academy of Ophthalmology (AAO) acknowledges the need for higher dosages or extended onset times in individuals with darker irises but does not specify precise dosing recommendations (Wallace *et al*., 2018). Given the lack of national guidelines and population-specific research, this study aims to evaluate the efficacy and safety of cyclopentolate and tropicamide in Indonesian children, optimising dosing regimens to enhance the accuracy of refractive assessments and improve clinical outcomes in paediatric ophthalmology.

## Methods

### Study design and subject selection

The study was conducted at RSCM Kirana Eye Clinic from June to August 2024 as a randomised, double-blind, controlled clinical trial to compare the residual accommodation amplitude at 20, 30 and 40 min following the administration of two instillations of either tropicamide 1% or cyclopentolate 1% (control), with a 5-min interval between doses. Participants were recruited using the consecutive sampling method from patients attending the paediatric outpatient clinic for refraction examination. Following informed consent, each participant received both cycloplegic agents in two separate sessions, spaced one week apart. Randomisation was performed using a true random number generator (random.org) to determine the order of cycloplegic administration. Participants assigned number 1 received cyclopentolate 1% as their initial agent, while those assigned number 2 received tropicamide 1% first.

Eligible participants included children aged 9–12 years with brown irises prior to pharmacologic dilation. Parents or guardians who had provided written informed consent and the participants who had provided assent, after receiving information regarding potential blurriness following cycloplegic instillation. Exclusion criteria encompassed the presence of active ocular infections, narrow anterior chamber angles, abnormal fundus reflexes, a history of neurological or cardiac disorders, ocular conditions other than refractive errors or strabismus, prior ocular surgery, developmental disorders, syndromic conditions, or documented hypersensitivity to the study medications. Additionally, children exhibiting uncooperative behaviour during examinations, an inability to maintain fixation on the autorefractor target, or refractive measurement discrepancies exceeding 0.5D across three consecutive readings in the same eye were excluded. Participants who failed to attend the second visit, withdrew consent before study completion, or had incomplete ocular examination records were classified as dropouts.

### Procedure

At the beginning of each session, one drop of 0.5% tetracaine as a topical anaesthetic agent, was instilled in each eye, starting with the right eye followed by the left, with a 1-min interval between eyes. One minute after tetracaine instillation, the first drop of the assigned cycloplegic agent (either 1% tropicamide or 1% cyclopentolate) was administered, followed 5 min later by a second drop of the same cycloplegic agent according to the randomisation schedule. A timing variation of ±1 min between instillation and autorefractor measurement was allowed. After the second cycloplegic instillation, participants waited 20 min before the second autorefractor measurement to assess residual accommodation and refractive correction. Additional measurements were conducted at 30 and 40 min post-instillation following the same procedure. To reduce blurred vision from the mydriatic effect after the final accommodation measurement, one drop of 2% pilocarpine hydrochloride was instilled in each eye. Both the study participants and the statistician responsible for data analysis were masked to the type of cycloplegic agent used to ensure masking and minimise bias.

To mitigate systemic absorption of the cycloplegic agents, nasal punctal occlusion was performed during each instillation. This involved gently pressing on the inner corner of each eye, at the location of the lacrimal punctum, for approximately 1–2 min immediately after drop administration. By temporarily blocking tear drainage into the nasolacrimal duct, this technique reduces the amount of drug entering the systemic circulation, thereby minimizing potential systemic side effects while maintaining the ocular effect of the medication.

### Autorefractometry

Refractive and accommodative measurements were conducted using the closed-field Auto Refractometer/Keratometer (NIDEK ARK-1s) (*NIDEK Co., Ltd., Japan*) in a standardised, well-lit examination environment. To minimise accommodation bias, the initial measurement was obtained prior to visual acuity assessment. The device provides an accommodative stimulus based purely on optical blur cues, without physical target movement and therefore without contributions from disparity or looming. The accommodative demand corresponds to the optical power shift produced internally by the instrument. All measurements were performed by a trained technician, who was masked to the allocated cycloplegic agent. During the procedure, participants were instructed to position their chin and forehead on the autorefractor support while maintaining both eyes open and fixating on an internal target, in which the accommodative demand increased from minimal to maximal through internally generated optical blur cues, without any physical movement of the target. In this closed-field autorefractor, accommodative demand is generated by an internal optical system that alters the vergence of light reaching the eye, typically ranging from near-zero diopters (distance-equivalent viewing) to several dioptres of near demand, thereby stimulating accommodation exclusively via blur cues, without binocular disparity, motion, or looming effects. Subjects were required to sustain fixation on the target for 30 s to ensure measurement consistency. Corneal reflex alignment was adjusted on the display screen, and measurements were recorded by activating the device’s measurement function.

Accommodation amplitude was determined by obtaining consecutive measurements in the same eye until two readings with a difference of no more than 0.50 D were recorded, with a maximum of three consecutive attempts. The final accommodation amplitude was defined as the last of the two consecutive values meeting this criterion, rather than the average. Refractive status measurements corresponded to the accommodation amplitude data. Autorefractor assessments were conducted for each eye separately, beginning with the right eye followed by the left, at four specific time points: (1) baseline (prior to cycloplegia), (2) 20 min after the second instillation of the cycloplegic agent, (3) 30 min post-instillation and (4) 40 min post-instillation. All refractive and accommodative parameters, including minimum accommodation, maximum accommodation and accommodation amplitude, were recorded in dioptres (D). For the purpose of this study, cycloplegia was considered clinically effective when the residual amplitude of accommodation was reduced to less than 2.5 dioptres, in accordance with the threshold described by Bartlett and Jaanus (2008). A secondary sensitivity analysis applying a more conservative threshold (residual accommodation <1.0 dioptre) was also performed and is presented in Appendix 1. Data from the right and left eyes were analysed independently as separate samples to ensure statistical rigor.

### Safety monitoring

Adverse drug reactions were monitored through direct observation for up to 1 h following cycloplegic instillation. Additionally, parents or guardians were provided with an online reporting form, which they could use to document any adverse reactions at any time. To ensure comprehensive follow-up, the research team also conducted telephone interviews with parents or guardians within 24 h to 7 days after the administration of each cycloplegic agent, using a standardised questionnaire.

The reporting form included information on simple management strategies for mild adverse effects, contact details of the research team and the address of the study hospital for further inquiries. Participants received their final autorefractive measurements via a personalised link, along with guidance emphasising the necessity of undergoing a subjective refraction assessment and consulting an ophthalmologist before using the measurements to obtain corrective eyewear.

### Statistical analysis

All collected data were systematically recorded in a master dataset in Excel Spreadsheet and analysed using the Statistical Package for Social Sciences (SPSS) version 25.0. Demographic and clinical characteristics were summarised descriptively, with quantitative variables presented as mean ± standard deviation (SD) and categorical variables as absolute counts and percentages. The normality of continuous data distributions was assessed using the Kolmogorov–Smirnov test. Normally distributed data would be reported as mean values, whereas non-normally distributed data would be expressed as medians.

The comparative effectiveness of 1% tropicamide and 1% cyclopentolate across different age groups, refractive error severities and post-instillation time points were assessed using paired *t*-tests. In cases where normality assumptions are not met or the sample size is limited, the Wilcoxon signed-rank test was employed. For paired categorical outcomes, such as the analysis of adverse effects, the McNemar test was used. All statistical analyses were conducted with a 95% confidence interval and a significance threshold of p < 0.05. A clinically relevant difference in residual accommodative amplitude and refractive error between cycloplegic agents is defined as ≥0.50 D. Data from the right and left eyes were analysed independently to ensure precise evaluation.

### Ethical considerations

This study was conducted in accordance with the principles outlined in the Declaration of Helsinki and received ethical approval from the Health Research Ethics Committee of the Faculty of Medicine, Universitas Indonesia and Dr. Cipto Mangunkusumo National Hospital (HREC FMUI–CMH) (Ethical Approval Registration Number: KET-1040/UN2.F1/ETIK/PPM.00.02/2024). The research protocol ensured participant confidentiality and was designed to minimise any risks or discomfort, with all procedures clearly explained to participants and guardians prior to enrolment.

## Results

### Subjects characteristics

This study was a non-inferiority trial conducted at a single centre, the RSCM Kirana Eye Clinic, from October 9, 2024, to November 23, 2024. A total of 96 patients who presented to paediatric ophthalmology outpatient care for refraction examination met the inclusion criteria, with 10 excluded based on the exclusion criteria, resulting in 86 participants receiving the first instillation according to the randomised allocation. Within one week after the first instillation, 6 subjects did not return for follow-up, 10 withdrew from the study and 10 exhibited excessive variability in measured accommodative amplitude across three consecutive assessments. Consequently, 70 participants proceeded to the quantitative data analysis phase. Since data were analysed per eye, and each participant contributed two eyes, the initial sample size was 140 eyes. Following the last instillation and objective assessment, participants were followed up to evaluate any incidence of adverse events. However, parents of 10 participants could not be contacted, reducing the final sample size for quantitative analysis to 60 participants.

The demographic characteristics of the study population showed a median age of 9 years (range: 9–12 years). The gender distribution was approximately 1:2, with female participants comprising 62.86% of the sample. The median uncorrected visual acuity (UCVA) for all eyes (n = 140) was 0.30 (equivalent to 6/20), with a minimum of 0.02 (1/60) and a maximum of 1.00 (6/6). Normal to mild UCVA impairment accounted for nearly half (48.57%) of the eyes of participants. Refractive status was categorised into emmetropia, myopia, hypermetropia and astigmatism. Emmetropic eyes were observed in 26.43% of the sample, while mild myopia was the most prevalent refractive error (38.57%), followed by moderate (22.14%) and high myopic eyes (9.29%). Hypermetropic eye was rare in the study population, with 2.86% exhibiting mild hypermetropic eye and only 0.72% (one eye) presenting with moderate hypermetropia, while no case of high hypermetropic eye was observed. All participants had brown irises. These demographic and refractive characteristics are summarised in Table 1, which presents the distribution of age, gender, UCVA and refractive status across the study sample.

**Table 1:** Demographic characteristic of study population.


CHARACTERISTICS	FREQUENCY

**Age, median [min–max] (years)**	11 [9–12]

**Gender, *n*(%)**	

Male	26 (37.14)

Female	44 (62.86)

**Uncorrected visual acuity (UCVA), (*N* = 140)**	

UCVA, median [min–max] (decimal)	0.30 [0.02–1.00]

**Severity of Visual Impairment, n(%)**	

Normal–mild	68 (48.57)

Moderate	38 (27.14)

Severe	16 (11.43)

Blindness^a^	18 (12.86)

**Refractive status (N = 140)**	

**Emmetropic eyes**	37 (26.43)

**Myopic eyes**	

Mild	54 (38.57)

Moderate	31 (22.14)

Severe	13 (9.29)

**Hypermetropic eyes**	

Mild	4 (2.86)

Moderate	1 (0.72)

Severe	0 (0.00)


^a^Blindness refers to visual acuity worse than 3/60 in one eye according to WHO criteria. No participant had no light perception, and all retained some degree of vision in each eye.

The study randomised participants into two groups, where each participant contributed two eyes that were analysed separately. Group A (n = 72 eyes) received 1% cyclopentolate as the first instillation, followed by 1% tropicamide after a washout period, while Group B (n = 68 eyes) received 1% tropicamide first, followed by 1% cyclopentolate. The spherical equivalent (SE) refractive status did not differ significantly between the two groups (Wilcoxon test, *p* = 0.947). Although the median UCVA differed between Group A (0.23) and Group B (0.40), the overall range remained comparable (0.02–1.00). Emmetropic eyes constituted more than a quarter of each group with minimal difference (26.39% in Group A vs. 26.47% in Group B). Mild myopic eye was the predominant refractive error in both groups (27.78% in Group A and 50.00% in Group B), followed by moderate and high myopic eye. Hypermetropic eyes accounted for a small proportion of both groups. These demographic and refractive characteristics for Group A and Group B are summarized in Table 2, which details the distribution of refractive errors and UCVA across both groups.

**Table 2:** Demographic characteristic of randomized group.


CHARACTERISTICS	A (*n* = 72)	B (*n* = 68)

**Refractive error (SE), median [min–max]**	–2.13 [–11.13–0.74]	–1.25 [–12.62–4.50]

**Visual acuity**		

UCVA (decimal), median [min–max]	0.23 [0.02–1.00]	0.40 [0.02–1.00]

**Refractive status, *n*(%)**		

**Emmetropic eyes**	19 (26.39)	18 (26.47)

**Myopic eyes**		

Mild	20 (27.28)	17 (50.00)

Moderate	18 (25.00)	13 (18.06)

Severe	12 (16.67)	1 (1.39)

**Hypermetropic eyes**		

Mild	3 (4.17)	1 (1.39)

Moderate	0 (0.00)	1 (1.39)

Severe	0 (0.00)	0 (0.00)


SE, standard error; UCVA, uncorrected visual acuity.

### Cyclopentolate effectivity

The effectiveness of the two cycloplegic eye drops was evaluated using accommodative amplitude (AA) as the primary outcome measure. AA was assessed at four time points: before instillation and at 20, 30 and 40 min post-instillation. Measurements were performed using an autorefractor, with the final AA value determined as the last recorded measurement from at least two consecutive readings. Normality testing of the collected AA data indicated a non-normal distribution across all datasets; therefore, data were presented as median values with minimum and maximum ranges. Statistical comparisons between the two cycloplegic agents were conducted using the Wilcoxon test.

Statistical analysis revealed no significant differences in AA outcomes between the two eye drops at any measured time point (*p* > 0.05). Cycloplegic efficacy was defined as achieving an AA of ≤2.50 D post-instillation. To assess this, the proportion of eyes meeting the efficacy threshold at each time point was calculated and compared between the two groups. The results demonstrated no statistically significant difference in the proportion of effective responses (Ef) of each eye between the two cycloplegic agents at any time point post-instillation (*p* > 0.05), as shown in Table 3, which compares the proportion of eyes meeting the efficacy threshold for both cycloplegic agents across time points.

**Table 3:** Comparison of accommodative amplitude (AA) and proportion of effectiveness following cycloplegic instillation.


	CYC1% *n* = 72		TRP1% *n* = 68		*p*	*P* Ef
	
	AA (DIOPTRE)	Ef (%)	AA (DIOPTRE)	Ef (%)		

Prior instillation	1.50 [0.26–9.38]	70.71	1.65 [0.23–8.71]	69.29	0.539	0.827

20 min-post	0.47 [0.10–2.79]	100.00	0.47 [0.14–3.54]	98.57	0.617	0.343

30 min-post	0.44 [0.13–3.91]	99.29	0.48 [0.13–3.63]	99.29	0.250	0.450

40 min-post	0.43 [0.11–4.06]	97.14	0.50 [0.17–3.61]	98.57	0.321	1.000

**Refractive error (SE), mean ± SD**	–1.38 [–11.12–4.38]		–1.50 [–12.62–4.50]		0.947	


*p < 0.05; AA, accommodative amplitude; Ef, effectivity. SD, standard deviation; CYC1%, Cyclopentolate 1%; TRP1%, Tropicamide 1%. *p < 0.05.

This study also assessed the progression of accommodative amplitude (AA) over time by calculating the change in AA (ΔAA) at each post-instillation time point. ΔAA was determined by subtracting the pre-instillation AA (AA at 0 min) from the AA measured at 20, 30 and 40 min post-instillation, providing a quantification of AA variation over time. The analysis of AA progression post-instillation revealed no statistically significant differences between the two eye drops, as shown in Table 4, which presents the change in AA (ΔAA) at each post-instillation time point for both cycloplegic agents.

**Table 4:** Comparison of cycloplegic eye drops on the delta accommodative amplitude across time points.


	Δ ACCOMMODATIVE AMPLITUDE, MEDIAN [MIN–MAX.] (DIOPTRES (D))					

	DELTA 1-0	*p*	DELTA 2-0	*p*	DELTA 3-0	*p*

**CYC1%**	–0.92 [–8.87–1.03]	0.681	–0.98 [–9.06–0.89]	0.455	–0.99 [–8.81–2.21]	0.561
		
**TRP1%**	–1.12 [–7.69–1.98]		–1.03 [–7.67–0.55]		–1.13 [–8.31–0.82]	


Delta 1-0, (Acc 20 mins post-instillation – Acc prior instillation); Delta 2-0, (Acc 30 mins post-instillation – Acc prior instillation); Delta 3-0, Acc 40 mins post-instillation – Acc prior instillation. SD, standard deviation; CYC1%, Cyclopentolate 1%; TRP1%, Tropicamide 1%.

Further analysis was conducted based on the degree of myopia to determine whether the cycloplegic effect differed according to baseline refractive status. Hyperopic refractive status was excluded from the analysis due to an insufficient sample size. A statistically significant difference was observed only at the 20-min mark post-instillation, with cyclopentolate 1% demonstrating a higher median AA compared to tropicamide 1%. Table 5 presents the cycloplegic effect on accommodative amplitude at Acc 0 (prior instillation), Acc 1 (20-min post-instillation), Acc 2 (30-min post-instillation) and Acc 3 (40-min post-instillation).

**Table 5:** Comparison of cycloplegic effectiveness based on the degree of myopia.


	CYC1%	TRP1%	*p*

**Accommodative Mild Myopic Eyes, median [min–max]**			

Acc 0	1.36 [0.31–8.77]	1.31 [0.23–8.71]	0.111

Acc 1	0.51 [0.18–7.72]	0.42 [0.17–1.92]	0.018*

Acc 2	0.41 [0.15–3.91]	0.48 [0.13–1.76]	0.097

Acc 3	0.35 [0.11–3.91]	0.40 [0.17–1.71]	0.652

**Moderate-Severe Myopic Eyes, median [min–max]**			

Acc 0	1.41 [0.26–9.38]	1.55 [0.53–7.82]	0.589

Acc 1	0.59 [0.15–1.70]	0.61 [0.18–1.95]	0.731

Acc 2	0.60 [0.13–1.88]	0.63 [0.17–3.63]	0.791

Acc 3	0.57 [0.22–3.23]	0.62 [0.20–3.23]	0.548


SD, standard deviation; CYC1%, Cyclopentolate 1%; TRP1%, Tropicamide 1%. *p < 0.05.

### Adverse effects

To evaluate the adverse effects of cycloplegic eye drops, the parents and participants were given online forms to report any symptoms at any given time while telephone interviews with parents were also conducted to ensure the documentation of any symptoms or complaints potentially related to the intervention, along with their duration. The collected data were analysed thematically, categorising reported complaints into ocular, systemic and combined ocular-systemic effects. If clinical considerations warranted intervention, appropriate management was provided by the researchers.

**Figure 1:**
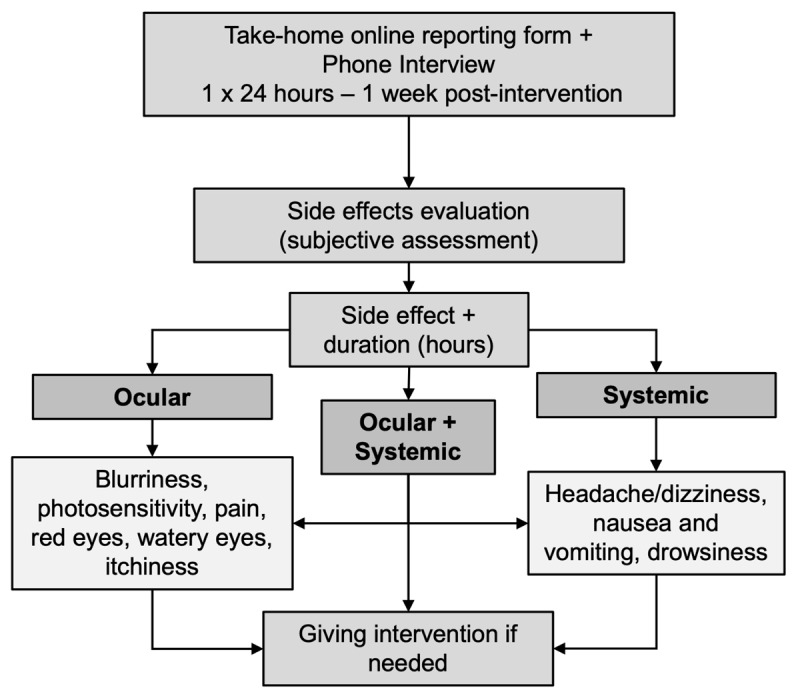
Adverse effects evaluation flowchart. Reported side effects are divided by ocular, systemic, or both.

The evaluation revealed that 46.67% of subjects reported no adverse effects following the instillation of cyclopentolate 1%, while a higher proportion (76.67%) remained asymptomatic after tropicamide 1%. This difference was statistically significant based on the McNemar test (p < 0.001).

The duration of adverse effects was presented as mean ± standard deviation (SD) to facilitate a more relevant comparison, irrespective of data distribution. On average, adverse effects lasted longer following cyclopentolate 1% administration than with tropicamide 1%. Regarding specific adverse effects, ocular complaints accounted for the majority (81.25%) of reported side effects from cyclopentolate 1%, while systemic and mixed ocular-systemic effects were each reported in 5.00% of cases. In contrast, all adverse effects associated with tropicamide 1% were purely ocular, with no systemic side effects reported. These findings are summarised in Table 6, which details the type and duration of adverse effects for both cycloplegic agents. No consistent order-related differences in adverse effect reporting were observed between the first and second study visits.

**Table 6:** Comparison of the proportion of adverse effects of cycloplegic eye drops and their duration.


	CYC1%, *n*(%)	TRP1%, *n*(%)	*p*

**No side effects**	28 (46.67)	46 (76.67)	<0.001*

**Side effects**	32 (53.33)	14 (23.33)	

**Duration, mean ± SD**,	15.63 ± 12.74	13.29 ± 9.94	

**Grouped duration, *n*(%)**	***n* = 32**	***n* = 14**	

<3 h	3 (9.4)	2 (14.29)	

3–12 h	9 (28.1)	4 (28.57)	

12–24 h	18 (56.3)	7 (50.00)	

**>** 24 h	2 (6.25)	1 (7.14)	


SD, standard deviation; CYC1%, Cyclopentolate 1%; TRP1%, Tropicamide 1%.

Among categorised durations as shown in Table 7, most subjects reported experiencing adverse effects for 12–24 h in both groups (56.3% for cyclopentolate 1% vs. 50.00% for tropicamide 1%), followed by 3–12 h, <3 h and >24 h. Two subjects (6.25% of those experiencing side effects, 2.86% of the total sample) reported adverse effects lasting beyond 24 h after cyclopentolate 1%, compared to only one subject (7.14% of those experiencing side effects, 1.43% of the total sample) after tropicamide 1%.

**Table 7:** Proportion of each adverse effect reported by intervention and its duration.


	CYC1%		TRP1%	
	
SIDE EFFECT	*n*(%)	DURATION, MEAN ± SD (h)	*n*(%)	DURATION, MEAN ± SD (h)

**Ocular**	**26 (43.33)**	**11.93 ± 10.19**	**14 (23.33)**	**13.29 ± 9.94**

Blurriness	13 (21.67)	9.42 ± 8.01	8 (13.33)	14.38 ± 8.21

Blurriness, photosensitive	7 (11.67)	17.86 ± 8.57	2 (3.33)	27.00 ± 4.24

Blurriness, photosensitive and other non-systemic complaint	2 (3.33)	24.00 ± 16.97	0 (0.00)	–

Blurriness and other non-systemic complaint	1 (1.67)	12.00	0 (0.00)	–

Itchiness	1 (1.67)	4.00		

Red eye	0 (0.00)	–	1 (1.67)	12.00

Pain	2 (3.33)	3.00 ± 0.00	3 (5.00)	1.67 ± 1.15

**Ocular + systemic**	**3 (5.00)**	**40.00 ± 13.86**	**0 (0.00)**	–

Headache, dizziness	2 (3.33)	36.00 ± 16.97		

Headache, dizziness, nausea	1 (1.67)	48.00		

**Systemic**	**3 (5.00)**	**11.17 ± 11.14**	**0 (0.00)**	–

Headache, dizziness	2 (3.33)	14.75 ± 13.08		

Drowsiness	1 (1.67)	4.00		

**Intervention-required side effects**	1 (3.33)		0 (0.00)	


SD, standard deviation; CYC1%, Cyclopentolate 1%; TRP1%, Tropicamide 1%. *p < 0.05.

The most frequently reported adverse effect following both cyclopentolate 1% and tropicamide 1% administration was blurred vision. In the cyclopentolate 1% group, the second most common complaint was a combination of blurred vision and photophobia, whereas in the tropicamide 1% group, eye discomfort upon instillation was more frequently reported than in the cyclopentolate group. Additionally, one subject reported eye redness following tropicamide 1%, while no such cases were observed after cyclopentolate 1% administration.

Among all study participants, only one subject (3.33% in the cyclopentolate group) required intervention for an adverse effect. Adverse effects lasting more than 24 h included systemic symptoms such as nausea, vomiting and dizziness following cyclopentolate 1%, as well as prolonged blurred vision after tropicamide 1%. One case of systemic symptoms in the cyclopentolate group was managed with carbachol eye drops.

## Discussion

Cycloplegic refraction is the gold standard for accurately diagnosing refractive errors in children, particularly to prevent amblyopia and impaired stereoscopic vision. However, studies on cycloplegic effectiveness in children with dark irises remain inconclusive, with no prior research on Indonesian children.

In this study, myopia was the most common refractive status, aligning with Al-Thawabieh *et al*. (2024). However, significant cycloplegic differences in hyperopia (*p* = 0.001; 95% CI: 0.104–0.396) and mixed refractive errors (*p* = 0.001; 95% CI: 0.136–0.494) but not in myopia was also reported (Yazdani *et al*., 2018). Similarly, another study also found greater cycloplegic effectiveness of cyclopentolate over tropicamide in hyperopia and mixed refractive groups (Pi *et al*., 2011). All participants also had brown irises, consistent with Indonesian ethnicity. While melanin is known to reduce cycloplegic effectiveness by binding muscarinic antagonists, a study has suggested that skin pigmentation may be a stronger factor (van Minderhout *et al*., 2022). This study did not stratify participants by skin tone, but pigmentation differences in Indonesia are generally minor.

Residual accommodative amplitude (AA) was used to assess cycloplegic effectiveness, as it measures remaining ciliary muscle function. Using 2.5 D threshold (Bartlett and Jaanus, 2008), both agents achieved nearly 100% effectiveness at 20–30 min, with overall effectiveness >95%. Stricter criteria (<1.0 D) would yield a maximum effectiveness of 92.86% (cyclopentolate 1% at 40 min) (Appendix 1). Although the threshold of 2.50 D is generally accepted as indicating adequate cycloplegia, minimal residual accommodation below this level may still be clinically relevant. In myopic eyes, even a small residual accommodative response can contribute to blur, which may reflect accommodative spasm rather than true refractive error (Horwood, 2022; Papageorgiou *et al*., 2021; Shukla, 2020). However, in the context of objective autorefractor-based accommodative measurements in children, small residual responses below 2–3 D may also represent measurement variability, fixation instability, or blur-driven responses rather than persistent voluntary accommodation. For this reason, the 2.5 D threshold was used to reflect functional suppression of accommodation, while a stricter criterion was explored as a sensitivity analysis to assess robustness under more conservative clinical assumptions.

Autorefractors, such as the Nidek ARK-1s used in this study, provide an objective and standardized method for measuring AA, reducing examiner-dependent variability and improving efficiency in children with limited attention spans. Compared to conventional techniques like the RAF ruler, autorefractors offer greater practicality; however, the requirement for prolonged eye opening (30 s) posed challenges (Kanclerz *et al*., 2022). In cases where manual eyelid assistance was necessary, the potential impact on tear film stability remains unknown. A study by Kanclerz *et al*. (2022) reported significantly lower AA values obtained via autorefractors compared to the push-up and minus lens methods (p < 0.001), though methodological and age-related differences limit direct comparison. Given that AA declines with age, further research is required to validate the reliability of autorefractor-based AA measurements specifically in paediatric populations.

The absence of standardised protocols for paediatric AA measurement, as well as a universally accepted threshold for cycloplegic effectiveness, underscores the need for further investigation. While this study utilized Bartlett’s 2.5 D criterion (Bartlett and Jaanus, 2008), alternative thresholds, such as AA <1.0 D, yielded different effectiveness rates, suggesting that current definitions of optimal cycloplegia may require refinement. Future studies should focus on establishing objective benchmarks for cycloplegic response assessment, considering variations in refractive status, iris pigmentation and pharmacokinetic properties of cycloplegic agents in diverse paediatric populations.

This study found no clinically significant difference between Cyclopentolate and Tropicamide in inducing cycloplegia in children. However, Tropicamide 1% demonstrated a statistically significant effect at 40 min (ΔAcc_40_ – Acc_0_), although this difference was not clinically meaningful. These findings are consistent with previous research, which has reported comparable cycloplegic efficacy between the two agents, with a slight advantage for Cyclopentolate 1% (Duran and Cevher, 2023). This study provides further evidence supporting their similar effectiveness, though Tropicamide may offer a slight benefit due to its faster onset, particularly in individuals with brown irises.

The selection of 20 min as the initial evaluation point was based on previous studies indicating a delayed cycloplegic onset in darker irises due to pigment binding (Pei *et al*., 2021). While research on adults has shown peak cycloplegic effects at 10 min in light-coloured irises and 30–40 min in dark irises (Laojaroenwanit *et al*., 2016), the present study suggests that in children, cycloplegia stabilizes by 20 min. Further investigation is warranted to assess effectiveness at earlier time points and beyond 40 min to optimize clinical protocols. Such data could be valuable in determining the necessity of repeat instillation when refractive assessments are delayed, as well as in refining patient management strategies to minimise waiting times in paediatric refraction examinations.

Age has also been proposed as a factor influencing cycloplegic onset, with previous studies suggesting peak effects at 50 min in children and 30–40 min in adults with dark irises (Laojaroenwanit *et al*., 2016; Manny *et al*., 1993). However, research remains inconclusive, particularly in myopic children, the predominant demographic in this study. Additionally, due to the limited number of hyperopic subjects, analysis was restricted to myopic participants. A statistically significant difference was observed at 20 min, where Tropicamide 1% resulted in a lower median residual accommodative amplitude than Cyclopentolate 1%; however, this difference did not persist at subsequent time points, indicating that while Tropicamide has a more rapid onset, both agents ultimately achieve comparable cycloplegic efficacy. Further large-scale studies are necessary to elucidate the influence of age and refractive status on cycloplegic onset and effectiveness. Additionally, the range of accommodative amplitudes (AA) in our cohort also indicates that some children still exhibited relatively high residual accommodative amplitude (>3 D), suggesting incomplete cycloplegia. Reported contributing factors include iris pigmentation, younger age with stronger accommodative tone, pharmacologic aspects (drop concentration and timing, uncooperativeness and the timing of measurement relative to drug instillation (Lim *et al*., 2019). This further underscores the need for clinical vigilance, as some children may not achieve full cycloplegia even with standard protocols.

Blurred vision and photophobia were the most frequently reported adverse effects, both of which are expected outcomes of cycloplegia and mydriasis (Bartlett and Jaanus, 2008; Sani *et al*., 2016). In contrast, systemic adverse effects such as flushing, dry mouth, or central nervous system symptoms are uncommon and not typically expected, though they have been reported in rare cases, particularly in children or with higher drug concentrations (Aldamri *et al*., 2024; Hürmüzlü Közler, 2022). This study found that adverse effects were more frequently reported after Cyclopentolate 1% instillation compared to Tropicamide 1% (53.33% vs. 23.33%; *p* < 0.001). These rates were higher than those in previous studies, particularly for Cyclopentolate. However, no severe systemic effects, such as ataxia, dysarthria, psychosis, or delirium, were observed. Given the relatively small sample size, these findings should be interpreted cautiously when assessing the overall safety profile of cycloplegic agents.

The most commonly reported symptoms were blurred vision and photophobia, which represent the expected pharmacological effects of cycloplegia and mydriasis rather than adverse side effects. This results from ciliary muscle and iris sphincter paralysis, which impairs near focus and increases light sensitivity due to pupil dilation (Jordan and Oatts, 2023). These effects were self-limiting and typically did not require intervention. In contrast, true adverse effects, particularly systemic symptoms, were only observed following Cyclopentolate instillation, likely due to absorption via the nasolacrimal duct and conjunctival mucosa. To mitigate systemic absorption, nasal punctal occlusion was performed in this study. This technique has been reported to reduce systemic side effects, enhance ocular drug penetration and prolong drug action (Rajeev *et al*., 2010), yet its implementation in routine clinical practice remains inconsistent.

Systemic side effects of Cyclopentolate, such as flushing, tachycardia, gastrointestinal intolerance, seizures and drowsiness, have been documented in prior research (Aldamri *et al*., 2024; Wakayama *et al*., 2018). In this study, the predominant systemic symptoms included headache, dizziness, nausea and drowsiness, likely due to Cyclopentolate’s higher lipophilicity, which facilitates blood–brain barrier penetration (Vaajanen and Vapaatalo, 2017). These effects were more frequently reported in younger children and those with lower BMI (van Minderhout *et al*., 2015). Meanwhile, Tropicamide 1% was associated with ocular redness in 5% of subjects, likely due to benzalkonium chloride-induced irritation or tear film destabilization, leading to corneal hyperosmolarity and nociceptor activation (Goldstein *et al*., 2022). Regarding duration, both agents exhibited prolonged effects (Cyclopentolate: 15.63 ± 12.74 h, Tropicamide: 13.29 ± 9.94 h). However, as these estimates were based on subjective recall rather than direct observation, further research is needed where clinicians are more involved in adverse effects evaluation and monitoring, therefore giving a more thorough and objective result to cycloplegia-related adverse effects.

This study’s primary strength lies in its direct measurement of accommodative amplitude (AA) post-cycloplegia, providing a precise evaluation of ciliary muscle suppression, which is an advancement over prior studies that relied on pupil diameter or spherical equivalent measurements. Notably, this is the first non-inferiority study comparing cycloplegic agents in Indonesian children, with existing data on dark-irised populations being limited. The use of an open-field autorefractor enhances accuracy and objectivity, reducing measurement bias, particularly in paediatric populations with communication challenges. Additionally, parental telephone interviews combined with online surveys improved adverse event reporting, enabling real-time clarification and higher response rates.

However, several limitations must be considered. The first measurement at 20 min post-instillation may not fully capture the onset dynamics of cycloplegia, potentially underestimating delayed effects in dark-irised populations. The lack of precise onset-time data limits understanding of pharmacodynamic variations, which is crucial for optimising clinical refraction timing. Moreover, reliance on parental recall introduces potential reporting bias, and the inclusion of adjunctive agents like tetracaine and pilocarpine complicates the attribution of adverse effects. These factors highlight the need for future studies with direct observational monitoring and controlled pharmacokinetic assessments to refine the evaluation of cycloplegia efficacy and safety in paediatric populations.

## Conclusion

Tropicamide 1% is non-inferior to cyclopentolate 1% in achieving effective cycloplegia in Indonesian children aged 9–12 years old while exhibiting a more favourable safety profile. Cyclopentolate 1% remains more potent but is associated with a higher incidence of adverse effects. Further research with larger sample sizes is needed to refine dosing strategies and optimize clinical applications in diverse paediatric populations.

## Appendix

**Appendix 1:** Effectivity analysis using 1.0 dioptre cut-off.

		CYC1% n = 72		TRP1% n = 68		P	P Ef

		AA(DIOPTRE)	Ef(%)	AA(DIOPTRE)	Ef (%)		

Prior instillation	OD	1.39 [0.31 – 8.76]	28,57	1,78 [0.23 – 8.71]	24.29	0.044	1.000

	OS	1.61 [0.26 – 9.38]	25.71	1.51 [0.61 – 8.65]	21.43	0.339	1.000

	ODS	1.50 [0.26 – 9.38]	27.14	1.65 [0.23 – 8.71]	22.86	0.539	1.000

20 minute-post	OD	0.44 [0.14 – 2.79]	90.00	0.52 [0.17 – 1.92]	87.14	0.235	0.648

	OS	0.51 [0.10 – 2.13]	90.00	0.45 [0.14 – 3.54]	87.14	0.663	0.664

	ODS	0.47 [0.10 – 2.79]	90.00	0.47 [0.14 – 3.54]	87.14	0.617	0.429

30 minutes-post	OD	0.46 [0.13 – 2.10]	85.71	0.51 [0.13 – 1.76]	88.57	0.554	0.774

	OS	0.42 [0.14 – 3.91]	91.43	0.48 [0.15 – 3.63]	88.57	0.33	0.804

	ODS	0.44 [0.13 – 3.91]	88.57	0.13 – 3.63]	88.57	0.250	0.571

40 minutes-post	OD	0.45 [0.11 – 4.06]	88.57	0.49 [0.19 – 1.80]	88.57	0.888	0.791

	OS	0.41 [0.17 – 3.23]	92.86	0.50 [0.17 – 3.61]	91.43	0.21	0.774

	ODS	0.43 [0.11 – 4.06]	90.71	0.50 [0.17 – 3.61]	90.00	0.321	1.000

*p < 0.05; AA, accommodative amplitude; Ef, effectivity. SD, standard deviation; CYC1%, Cyclopentolate 1%; TRP1%, Tropicamide 1%. *p < 0.05.
